# Differential assembly and functional roles of bacterial communities in coniferous and mixed conifer-broadleaf forest soils

**DOI:** 10.1128/msphere.00627-25

**Published:** 2026-03-06

**Authors:** Dexing Chen, Ziyang Zhang, Shunfen Wang, Wenhui Li, Yimin He, Wenyu Zhang, Weiwei Sun, Mingjiu Chen, Shuangquan Zou, Xin Qian

**Affiliations:** 1 Fujian Colleges and Universities Engineering Research Institute of Conservation and Utilization of Natural Bioresources, College of Forestry, Fujian Agriculture and Forestry University, Fuzhou, China; 2 Key Laboratory of National Forestry and Grassland Administration for Orchid Conservation and Utilization at Colleage of Landscape Architecture, Fujian Agriculture and Forestry University 12449, Fuzhou, China; 3 College of Forestry, Fujian Agriculture and Forestry University 12449, Fuzhou, China; 4 Gufeng State-owned Forest Farm of Pingnan County, Pingnan, China; University of South Africa, Florida, Johannesburg, Gauteng, South Africa

**Keywords:** soil bacterial communities, coniferous and mixed forests, microbial diversity, co-occurrence networks, community assembly

## Abstract

Forest soils harbor a diverse array of bacteria that play a crucial role in nutrient cycling. However, the differential effects of coniferous versus mixed conifer-broadleaf forests on the distribution of both abundant and rare bacterial taxa remain poorly understood. In this study, we integrated 16S rRNA gene amplicon sequencing with metagenomic shotgun sequencing to conduct a comparative analysis of soil bacterial communities in a conifer plantation and an adjacent mixed conifer-broadleaf forest, specifically examining their community structure, assembly mechanisms, co-occurrence networks, and functional potential. Both abundant and rare taxa showed significant differences in community composition between the two forest types. Soil pH and organic matter content significantly influenced the total and abundant bacterial communities, while available phosphorus and potassium were key determinants of rare community composition. Co-occurrence network analysis revealed that abundant communities formed highly clustered, simplified networks, contrasting with more fragmented and keystone-rich networks in rare communities. Null model analyses indicated that community assembly was largely driven by stochastic processes, with ecological drift accounting for about 80% of the variation in total and rare communities, and dispersal limitation explaining nearly 72% of the variation in abundant communities. Functional predictions indicated that bacterial communities in mixed forests were enriched in pathways linked to glycosylation, carbohydrate degradation, and nitrogen fixation, while coniferous forests favored pathways related to autophagy, signaling, and stress responses. This study highlights the complementary roles of abundant and rare bacterial taxa in forest soil ecosystems and underscores the importance of preserving mixed forests to sustain microbial functional diversity.

**IMPORTANCE:**

Forest soils host a complex web of common and rare bacteria that quietly regulate nutrient cycles. By comparing pure conifer stands with mixed conifer-broadleaf forests, we found that abundant species underpin essential functions while rarer microbes fill specialized niches. Acidity and nutrients strongly influence which bacteria thrive; mixed stands favored microbes that break down carbohydrates and fix nitrogen, whereas conifer soils supported organisms adapted to stress and nutrient-poor conditions. These findings emphasize the importance of preserving diverse forest ecosystems for soil health, carbon storage, and effective forest management strategies in climate change adaptation.

## INTRODUCTION

Forest ecosystems are central to global biodiversity and ecological functions, playing crucial roles in carbon storage, climate regulation, soil and water conservation, and biodiversity preservation. Soil bacterial communities, as essential components of these ecosystems, drive biogeochemical cycles, including those of carbon, nitrogen, and phosphorus, and support key ecological processes such as organic matter decomposition, nutrient cycling, soil structure formation, and fertility regulation (1, 2). Therefore, understanding the structure and function of soil bacterial communities is vital for preserving the sustainability of forest ecosystems.

Coniferous forests and conifer-broadleaf mixed forests are the two dominant forest types in the temperate and boreal regions of the Northern Hemisphere. Although coniferous forests are characterized by high carbon storage capacity and substantial timber production value, their relatively low biodiversity and limited resilience to pests, diseases, and environmental disturbances constrain their overall ecological service functions (3). Introducing broadleaf species into these systems gives rise to conifer-broadleaf mixed forests, which enhance ecosystem diversity and stability by supporting more complex ecological processes, including water conservation and soil protection. However, these mixed forests also bring additional challenges in terms of management and cultivation (4). While previous studies have examined microbial characteristics within these forest types, the contrasting ecological contexts of pure coniferous versus mixed forests provide an ideal framework to explore differences in soil microbial community assembly— particularly the functional roles and interactions of abundant and rare bacterial taxa (5). A comparative investigation of soil bacterial community structure and function across these ecosystems will therefore help clarify how forest type shapes microbial processes and inform forest management and conservation strategies.

Microbial communities in various environments typically consist of a few abundant taxa and a greater number of rare taxa. Recent research has emphasized the functional differences between these groups and increasingly recognized the ecological importance of rare species (6, 7). Abundant microbial communities, particularly those dominated by bacteria, often play a key role in critical ecological functions, such as organic matter decomposition, nutrient cycling, and gas exchange (8). For instance, nitrogen-fixing, nitrifying, and denitrifying bacteria are core participants in the carbon and nitrogen cycles, and their high abundance in bacterial communities is closely linked to soil nutrient content (9). In contrast, while rare bacteria are less abundant in soil, they perform indispensable roles in maintaining ecosystem stability and function under specific environmental conditions. For example, rare taxa like *Arthrobacter* species have been shown to thrive in nutrient-poor soils, where they play crucial roles in breaking down recalcitrant organic matter and facilitating the availability of nutrients to other microorganisms (10). These rare taxa are often adapted to survive in nutrient-poor or extreme soil environments and contribute to specialized nutrient transformation processes, thus supporting the overall diversity and resilience of ecosystems.

The assembly process of microbial communities has long been a central topic in microbial ecology. In the context of forest soils, community assembly refers to the integrated processes of species selection, competition, and interactions within the soil environment, where environmental factors such as soil pH, nutrient availability, moisture, and organic matter content influence microbial colonization and establishment (11). Recent studies have highlighted that the formation of microbial communities is not solely shaped by environmental factors and stochastic processes but also by complex ecological interactions among species within the community. These interactions, including symbiosis, competition, predation, and mutualism, collectively determine the stability and functional characteristics of community structure (12). Moreover, gene-level functional analyses have provided valuable insights into the ecological roles of microbial communities, revealing potential functional relationships between species within the community (13). In parallel, microbial coexistence networks have emerged as powerful tools for characterizing microbial interactions and are widely applied in community studies across diverse ecosystems (14). By constructing such networks, researchers can identify cooperative, competitive, and antagonistic relationships among microbial populations, thereby deepening our understanding of the mechanisms that sustain community stability and diversity (15). These studies suggest that cooperative interactions between abundant and rare bacteria contribute positively to community stability, thereby optimizing ecosystem functions.

However, despite the widespread use of microbial coexistence networks and the concept of community assembly across various ecosystems, there is still a lack of detailed research on the differences in assembly processes between abundant and rare bacterial communities in forest ecosystems, particularly in soils from different forest types (16). Forest soils, with their high heterogeneity and variability, offer an ideal environment for exploring these microbial community differences. Soils from different forest types exhibit significant variations in physicochemical properties, such as pH, nutrient levels (e.g., nitrogen and phosphorus content), and soil moisture. These variations can exert distinct ecological selection pressures, profoundly influencing the structure and function of microbial communities (17). For instance, in extreme soil environments, rare bacteria may rely on specialized adaptive mechanisms to become key functional regulators, thus maintaining community stability and ecological balance (18). Therefore, investigating the distribution, functional differences, and interactions between abundant and rare bacterial communities in soils from different forest types, along with their relationships to soil factors, is essential for understanding the mechanisms behind microbial community assembly and their ecological functions.

This study was conducted at the Pingnan Gufeng State-owned Forest Farm, located in southeastern China, which has a cultivation history exceeding 6 decades since its establishment in 1958. The forest primarily comprises coniferous mixed forests and conifer-broadleaf mixed forests. This distinctive site provides an ideal opportunity to investigate the structure, composition, and function of soil microbial communities within these two forest types. The objectives of this study are (i) to explore the differences in bacterial community assembly, specifically focusing on the roles of abundant and rare taxa in the two forest soil environments, and (ii) to identify key functional pathways in bacterial communities and assess how forest type (coniferous vs mixed conifer-broadleaf) shapes these microbial functional potentials.

## MATERIALS AND METHODS

### Study area

The investigation was carried out at the Pingnan Gufeng Forest Farm (26°54′N, 118°58′E), located in Fujian Province, southeastern China. The experimental site is situated on a north-facing slope at elevations ranging from 965 to 1,005 m above sea level. The region experiences a subtropical monsoon mountain climate, characterized by a frost-free period of approximately 230 days, an annual sunshine duration of 1,501.5 hours, and a mean annual precipitation of 2,034.9 mm distributed across 226 days. The mean annual temperature varies between 14.6°C and 19°C, with midsummer averages of 24°C and recorded extremes ranging from −6.4°C to 32.2°C. The dominant soil type is neutral karst yellow-red earth.

The study focused on two 31-year-old forest stands: a coniferous forest dominated by Masson pine (*Pinus massoniana*) and Chinese fir (*Cunninghamia lanceolata*), and a conifer-broadleaf mixed forest consisting of Chinese fir, Masson pine, and *Quercus elevaticostata*. In the mixed forest, the species composition ratio was 4:15:6, with a stand density of 2,501 trees per hectare, a canopy density of 0.75, and a stand volume of 476.268 m³ ha⁻¹. The coniferous forest had a Masson pine-to-Chinese fir ratio of 1:10, a stand density of 3,301 trees per hectare, a canopy density of 0.65, and a stand volume of 467.553 m³ ha⁻¹.

### Experimental design and soil property analysis

For each forest type, three replicate 10 × 10 m plots were established, with a minimum inter-plot distance of 50 m to reduce spatial autocorrelation. The plots were selected to represent the typical ecological conditions of each forest type, considering variations in tree density, species composition, and soil properties. By establishing multiple plots, we aimed to capture the natural variability within each forest type, ensuring that the findings were not biased by local anomalies. Within each plot, understory vegetation, species composition, and tree growth attributes were surveyed. Tree height, diameter at breast height, and canopy width were recorded for all individuals. Each plot was further divided into four 5 × 5 m subplots, from which three were randomly selected for soil sampling. This random selection ensures that the soil samples reflect the spatial heterogeneity of the forest floor.

Composite soil samples from the 0-20 cm depth were collected within each subplot by randomly selecting three points, and soils from these points were mixed to create a single composite sample. Each composite sample was divided into two parts: one was immediately placed in a cooler with ice packs and transported to the laboratory, where it was stored at -80°C for subsequent microbial DNA extraction; the other was air-dried for physicochemical analysis. Soil physicochemical properties included soil bulk density (SBD), soil organic matter (SOM), pH, available potassium (AK), total potassium (TK), available phosphorus (AP), total phosphorus (TP), and alkali-hydrolyzable nitrogen (AN). SBD was determined using the core method with undisturbed samples oven-dried at 105°C to constant weight. SOM was quantified via potassium permanganate oxidation. Soil pH was measured in a 1:2.5 (wt/vol) soil-water suspension using a calibrated pH meter. AK and TK were analyzed by flame photometry following extraction with ammonium acetate and digestion with nitric-perchloric acid, respectively. AP and TP were determined spectrophotometrically using the molybdenum blue method after appropriate extraction and digestion. AN was assessed using the alkaline diffusion method with sodium hydroxide. All analytical procedures adhered to the standard protocols described by Bao (19), ensuring methodological consistency and data accuracy.

### DNA extraction and gene amplification

Total genomic DNA was extracted from soil samples using the E.Z.N.A. Soil DNA Kit (Omega Bio-tek, USA). The quality and concentration of the extracted DNA were assessed by 1.0% agarose gel electrophoresis and quantified using a NanoDrop 2000 spectrophotometer (Thermo Scientific, USA). High-quality DNA samples were stored at −80°C for subsequent analysis.

The hypervariable V3-V4 region of the bacterial 16S rRNA gene was amplified with the primers 338F (5′-ACTCCTACGGGAGGCAGCA-3′) and 806R (5′-GGACTACHVGGGTWTCTAAT-3′). Polymerase chain reaction was performed under the following conditions: initial denaturation at 95°C for 5 minutes; 25 cycles of denaturation at 95°C for 30 seconds, annealing at 55°C for 30 seconds, and extension at 72°C for 45 seconds; followed by a final extension at 72°C for 7 minutes. Amplified products were purified using the AxyPrep DNA Gel Extraction Kit, quantified with the Quantus Fluorometer (Promega, USA), and used for library construction with the NEXTFLEX Rapid DNA-Seq Kit (Bioo Scientific, USA). High-throughput sequencing was conducted on the Illumina NovaSeq 6000 platform.

Raw sequencing data were generated in FASTQ format, containing both sequence reads and corresponding base quality scores (20). Low-quality reads were removed during quality control: reads containing ambiguous bases (N), exhibiting abnormal length, or with low average Phred quality scores were discarded. In addition, bases with low quality (e.g., Phred score <20) were trimmed, and reads shorter than a minimum length after trimming were excluded to ensure downstream reliability. Paired-end reads were then merged using FLASH software (21) with a minimum overlap requirement and mismatch constraint. Chimeric sequences were identified and removed prior to operational taxonomic unit (OTU) construction. High-quality sequences were clustered into OTUs based on 97% similarity. Representative sequences from each OTU were taxonomically classified using the SILVA database (v.138) and the RDP classifier (22).

To distinguish between abundant and rare bacterial taxa, relative abundance thresholds were applied following a modified framework established by Jiao et al. (23). OTUs with an abundance ≥0.1% across all samples were classified as “abundant,” while those with an abundance ≤0.01% were designated as “rare.”

### Metagenomic sequencing and bioinformatics analysis

To investigate differences in soil microbial functional potential between coniferous and conifer-broadleaf mixed forests, metagenomic sequencing was performed on six soil samples (three per forest type). Raw reads were preprocessed with FASTP to remove low-quality sequences and adapter contaminants (21). Quality-filtered reads were assembled *de novo* using MEGAHIT, and contigs with lengths ≥300 bp were retained for downstream analysis (24).

Open reading frames were predicted from assembled contigs using Prodigal, and those longer than 100 bp were translated into amino acid sequences (25). A non-redundant gene catalog was constructed by clustering the predicted genes with CD-HIT (26). Functional annotation was performed by aligning non-redundant gene sequences against the KEGG database using BLAST. Differential abundance analysis of metabolic pathways between forest types was conducted to identify ecologically significant functional traits.

### Statistical analysis

All statistical analyses were performed in R (version 4.5.0). α-Diversity was assessed using the Shannon index calculated with the “vegan” package (27), and group differences were tested using ANOVA followed by Tukey’s HSD post hoc comparisons. Differences in β-diversity were evaluated using principal coordinate analysis (PCoA) based on Bray-Curtis distances. PCoA was selected because it provides an explicit Euclidean representation of the original dissimilarity matrix and reports the proportion of variation explained by each ordination axis, facilitating interpretation and comparison of community differentiation among groups. The statistical significance of community separation among groups was tested using permutational multivariate analysis of variance implemented in “vegan” (28). Community composition was visualized by stacked bar plots at the phylum level.

Differential abundance analysis was conducted with the “DESeq2” package (29), which applies a negative binomial generalized linear model to identify taxa with significant changes in abundance between forest types. Volcano plots were generated to display significantly enriched or depleted taxa.

To assess the influence of environmental factors on microbial structure, Spearman correlation coefficients between soil physicochemical variables and microbial relative abundance were calculated and visualized as heatmaps using the “microeco” package (30). Mantel tests, implemented via “LinkET,” were used to evaluate correlations between environmental distance matrices and microbial Bray–Curtis dissimilarity matrices (31). Redundancy analysis (RDA) was performed with “vegan” to quantify the proportion of community variance explained by soil properties, and significance of the RDA model and axes was tested using permutation tests (32).

Bacterial co-occurrence networks were constructed using the “ggClusterNet” package (33) based on Spearman correlation (*P* > 0.7 and FDR-adjusted *P* < 0.05). Network topology indices—including node count, edge number, degree distribution, clustering coefficient, average path length, network density, and betweenness centrality—were calculated to describe network complexity and interaction patterns. The “psych” package was used to examine correlations between network-level metrics and soil environmental variables (34).

To infer the ecological processes governing community assembly, the null model-based iCAMP framework (35) was applied. β-Nearest taxon index (βNTI) and RCbray values were computed using the “NST” package to distinguish deterministic (homogenizing selection and variable selection) versus stochastic (homogenizing dispersal, dispersal limitation, and drift) processes following Stegen et al. (36).

Functional profiling of bacterial communities was performed using KEGG Orthology (KO) annotations from metagenomic data. Differential enrichment of metabolic pathways was identified through KO-based statistical testing, followed by Z-score normalization, and significance evaluated using Reporter score analysis (threshold > 1.96). Functional differences were visualized using bar and bubble plots.

## RESULTS

### Community structure of bacterial communities

A total of 1,584,307 high-quality bacterial sequences were obtained from all soil samples, which clustered into 3,079 OTUs. Based on established relative abundance thresholds, OTUs were categorized as either abundant or rare. The results indicate that although abundant bacterial OTUs constituted only 176 taxa, they accounted for 71.10% of the total microbial sequences. In contrast, the 2,194 rare bacterial OTUs represented merely 7.20% of the total sequences.

**Fig 1:**
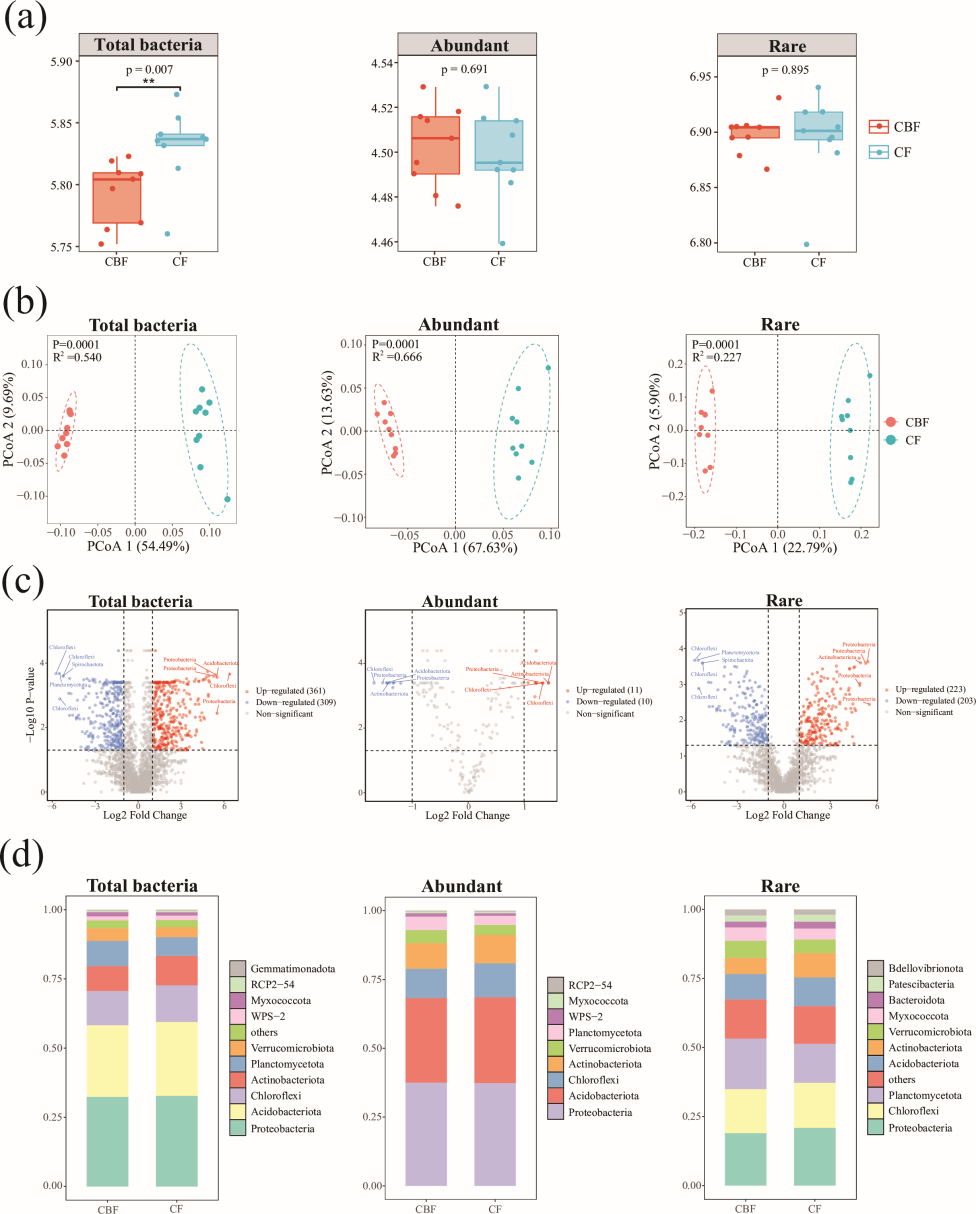
Comparative analysis of microbial community diversity and composition. (**a**) Differences in the α-diversity indices (Shannon index) of total bacterial communities, abundant bacterial communities, and rare bacterial communities in soil samples from coniferous forests and mixed conifer-broadleaf forests. (**b**) Unconstrained PCoA based on Bray-Curtis distance shows the differences in bacterial community composition between the two forest types. Ellipses represent the 95% confidence intervals for each sample type. Asterisks denote significance levels: * indicates *P* < 0.05, ** indicates *P* < 0.01, and *** indicates *P* < 0.001. “CBF” represents mixed conifer-broadleaf forests, and “CF” represents coniferous forests. (**c**) The volcano plot illustrates the enrichment and depletion of functional fungal OTUs in coniferous forests compared to mixed conifer-broadleaf forests. Each point represents an OTU, with red indicating upregulation and blue indicating downregulation. The *x*-axis represents log2 fold change (abundance change ratio), and the *y*-axis represents -log10 FDR values (statistical significance of abundance changes). The top 5 upregulated and downregulated OTUs are associated with their phylum information. (**d**) The stacked bar chart at the phylum level shows the composition of bacterial communities. Each layer in the chart represents the relative abundance of a specific phylum, with different colors indicating distinct bacterial phyla. The top 10 phyla are displayed, and other unlisted phyla are categorized as “others.”

Comparative analysis between forest types revealed that the Shannon diversity index of the total bacterial community was significantly higher in coniferous forests than in mixed coniferous-broadleaf forests (Fig. 1a). However, no significant differences were observed in the diversity of either abundant (*P* = 0.691) or rare (*P* = 0.895) subcommunities, suggesting that forest type influences overall bacterial diversity but not the diversity of specific abundance-based subgroups. PCoA based on Bray-Curtis distances indicated significant compositional divergence in soil bacterial communities between forest types (Fig. 1b; *P* < 0.001). Clear separations were observed among total, abundant, and rare communities. Rare taxa exhibited greater heterogeneity, as reflected by a lower *R*² value.

Volcano plot analysis (Fig. 1c) highlighted differential abundance of OTUs between forest types. In the total bacterial community, 361 OTUs were significantly upregulated and 309 downregulated, predominantly within Proteobacteria, Chloroflexi, and Acidobacteriota, indicating a substantial forest-type effect. Within the abundant subgroup, only 11 OTUs were upregulated and 10 downregulated, with minor shifts occurring mainly in Actinobacteriota, Acidobacteriota, and Chloroflexi. In contrast, the rare subcommunity exhibited more pronounced changes, with 223 OTUs upregulated and 203 downregulated, largely within Proteobacteria, Actinobacteriota, and Chloroflexi. These results underscore the differential responsiveness of rare taxa to forest type and their potential functional importance. At the phylum level (Fig. 1d), Proteobacteria, Chloroflexi, and Acidobacteriota dominated both the total and abundant bacterial communities in both forest types. The rare bacterial community demonstrated higher phylogenetic diversity; although Proteobacteria and Chloroflexi remained prevalent, numerous additional phyla were also present.

**Fig 2:**
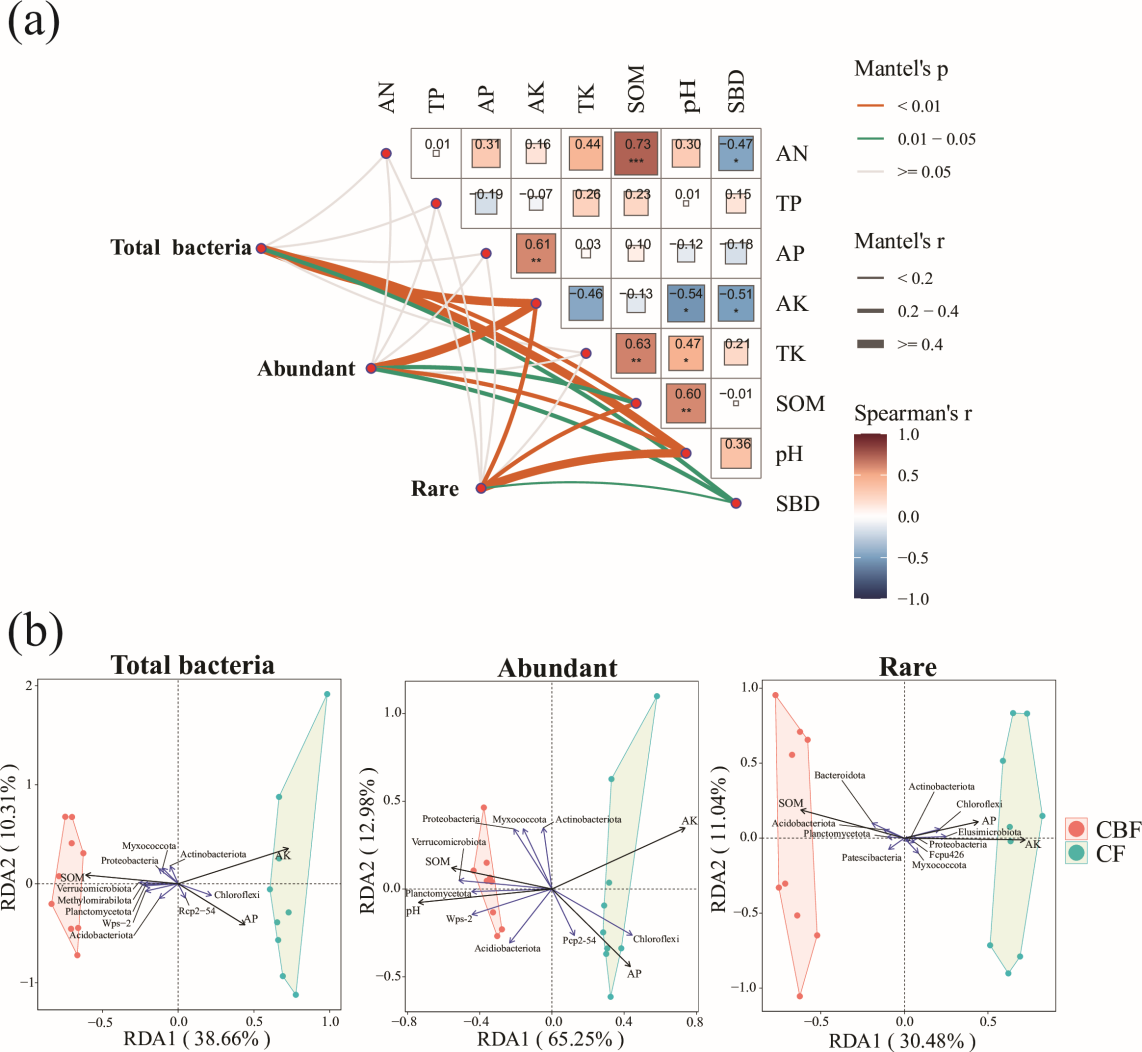
Correlation analysis between environmental factors and bacterial communities. (**a**) Mantel test assessing the correlation between environmental factors and bacterial community composition. Each cell in the heatmap represents the Pearson correlation coefficient (*r*) between a specific environmental factor and the bacterial community, with color intensity reflecting the strength of the correlation. Red indicates a positive correlation, blue indicates a negative correlation, and deeper colors represent stronger correlations. The significance of the correlation is indicated by asterisks: *P < 0.05, **P < 0.01, ***P < 0.001. (**b**) Redundancy analysis (RDA) plot at the phylum level showing the impact of environmental factors on bacterial community composition. Arrows represent the influence of environmental factors, with their length reflecting the strength of the effect. Sample positions indicate the influence of environmental factors on community structure.

Significant variations were observed in soil physicochemical properties between forest types. Soil pH and SOM were significantly higher in mixed conifer-broadleaf forests than in coniferous forests, while AK was significantly lower in mixed forests (Fig. S1). Mantel tests revealed significant positive correlations between SOM and both total and abundant bacterial communities. AP showed weak correlations with total and rare communities but exhibited negative associations with abundant taxa. SOM was identified as the strongest consistent predictor across all community types (Fig. 2a). Redundancy analysis demonstrated that SOM exerted the strongest influence on total and abundant communities, particularly in mixed conifer-broadleaf forests (CBF). AP significantly affected total and rare community structures, with the most pronounced effects in coniferous forests (CF). AK was also found to shape abundant community composition, especially in CF (Fig. 2b).

### Co-occurrence networks

**Fig 3:**
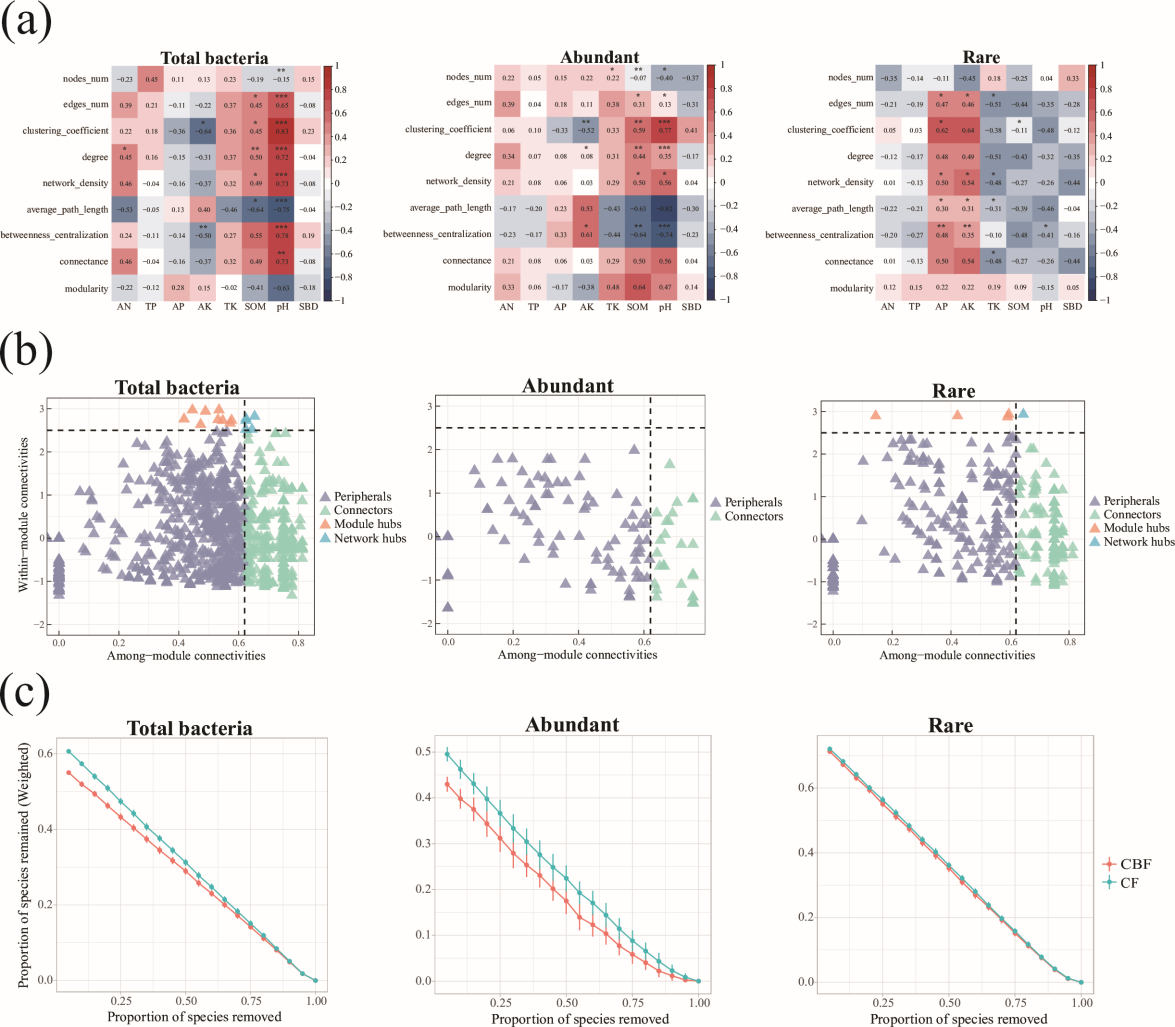
Bacterial community network topology and stability analysis. (**a**) Correlation heatmap between environmental factors and bacterial community topology properties, showing the Pearson correlation coefficients between various environmental factors and bacterial community topology attributes. Red indicates a positive correlation, blue indicates a negative correlation, and the intensity of the color reflects the strength of the correlation. (**b**) ZiPi plot, with the *x*-axis representing inter-module connectivity (Zi) and the *y*-axis representing intra-module connectivity (Pi). Nodes are colored to distinguish between the three community types, and the quadrants divide the nodes into four types: peripheral nodes, connectors, module hubs, and network hubs. (**c**) Robustness analysis plot showing the effect of sequential species removal (*x*-axis) on community stability (*y*-axis, remaining connectivity ratio), comparing the disturbance resistance of the total, abundant, and rare communities.

Distinct topological features were observed among the co-occurrence networks of total, abundant, and rare bacterial communities (Table S1). The total bacterial community exhibited high node connectivity and network redundancy, suggesting considerable functional stability. Elevated connectivity and local clustering coefficients indicated efficient information transfer, with both pH and SOM exerting positive regulatory effects on these topological properties (Fig. 3a), underscoring their role in enhancing community stability and functional coordination.

In contrast, the abundant bacterial community displayed a more streamlined network architecture, characterized by nodes predominantly located in peripheral and connector regions, yet maintaining strong local clustering (Fig. 3b). AK negatively influenced clustering and information transfer, implying a suppressive effect on network optimization. Conversely, SOM supported structural organization within this community. Robustness analysis indicated rapid disintegration following node removal, reflecting structural fragility under perturbation (Fig. 3c).

The rare bacterial community demonstrated reduced connectivity and lower efficiency in information transfer. Although AP and AK contributed positively to network expansion and connectivity, these effects were outweighed by the inhibitory roles of TK, SOM, and pH on clustering and transfer efficiency (Fig. 3a), indicating limited ecological adaptability and heightened susceptibility to environmental fluctuation. Despite low abundance, certain rare taxa occupied hub and connector positions (Fig. 3b), suggesting potential functional keystone roles. Nonetheless, robustness analysis confirmed higher sensitivity to disturbance and insufficient functional redundancy (Fig. 3c).

### Community assembly processes

**Fig 4:**
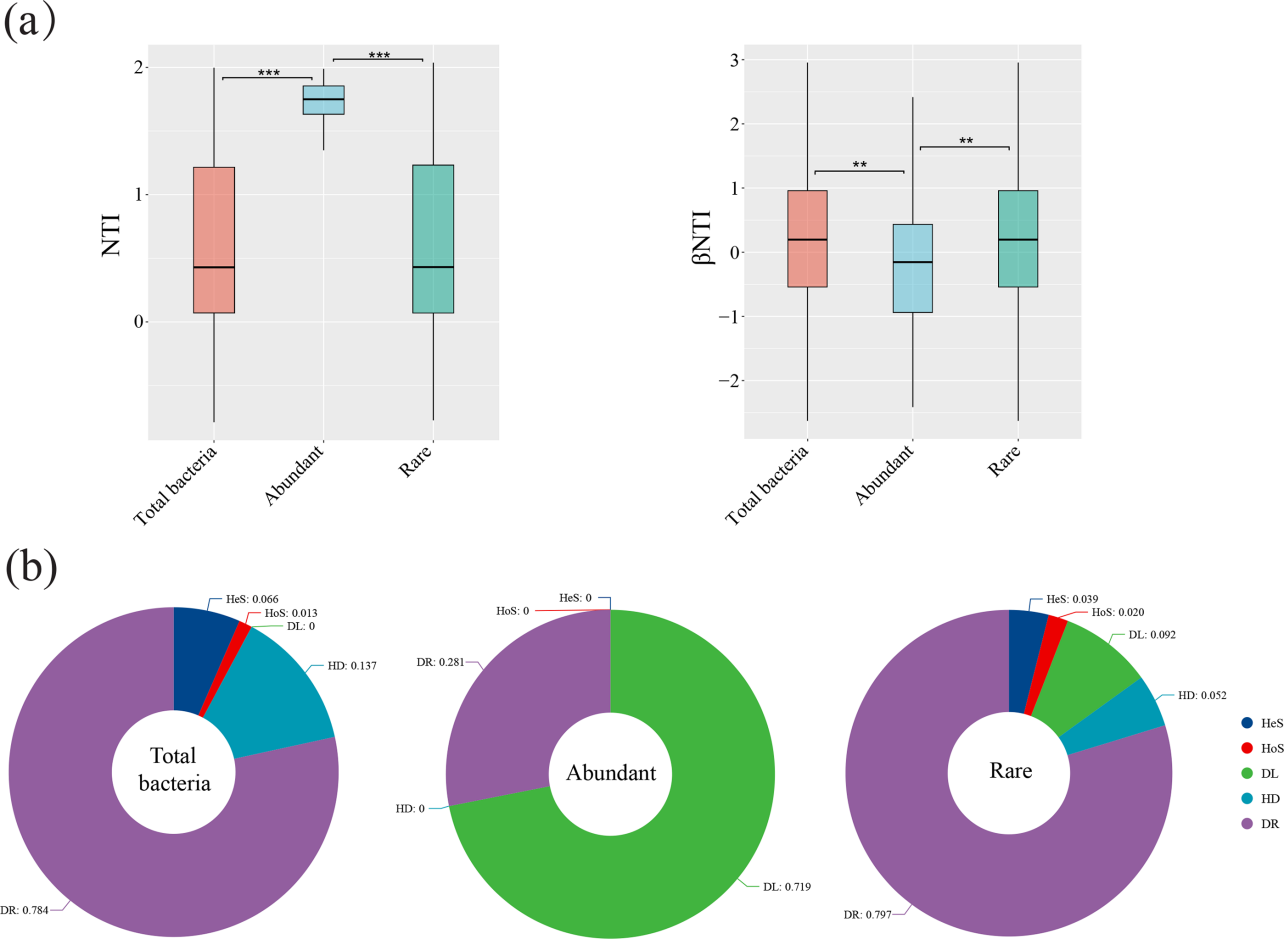
Phylogenetic indices and community assembly. (**a**) NTI and βNTI boxplots. The *x*-axis represents community types, and the *y*-axis represents NTI and βNTI values. NTI (nearest taxon index) measures phylogenetic clustering within a community; NTI > +2 indicates significant phylogenetic clustering (environmental filtering), NTI < –2 indicates phylogenetic overdispersion (possible competitive exclusion), and |NTI| ≤ 2 suggests that stochastic processes (e.g., drift or random dispersal) dominate community assembly. βNTI (Beta nearest taxon index) measures phylogenetic turnover between communities; βNTI > +2 indicates heterogeneous selection (deterministic), βNTI < –2 indicates homogeneous selection, and |βNTI| ≤ 2 indicates stochastic processes such as dispersal limitation or drift. Significance is indicated by asterisks: **P < 0.01, ***P < 0.001. (**b**) Process decomposition pie chart of community assembly. Colors represent heterogenizing selection (HeS), homogenizing selection (HoS), dispersal limitation (DL), homogenizing dispersal (HD), and ecological drift (DR).

The assembly of bacterial communities was predominantly governed by stochastic processes. Values of the NTI across communities ranged from –2 to +2, indicating an absence of strong phylogenetic clustering or overdispersion at the overall community level. Notably, however, the abundant bacterial community exhibited a significantly higher mean NTI compared to both the total and rare communities, suggesting weak phylogenetic clustering and a potential influence of selective pressure on high-abundance taxa (Fig. 4a). This pattern was corroborated by βNTI analysis. Most pairwise βNTI values for the total and rare communities fell within the range of ±2, consistent with dominance by stochastic assembly. In contrast, the abundant community showed a left-skewed βNTI distribution with lower values, indicating greater phylogenetic similarity among its samples and a stronger signature of deterministic selection (Fig. 4a).

Decomposition of assembly processes based on βNTI and RCbray metrics further supported these patterns (Fig. 4b). For the total bacterial community, ecological drift accounted for 78.4% of β-diversity, with homogenizing dispersal and heterogenizing selection contributing 13.7% and 6.5%, respectively. Similarly, in the rare community, drift remained the dominant process (79.7%), while all forms of selection together constituted less than 6%. In stark contrast, dispersal limitation explained 71.9% of the assembly in the abundant community, with drift accounting for the remaining 28.1%. These results indicate that high-abundance taxa are predominantly structured by spatial constraints, under modest environmental filtering—a conclusion consistent with their elevated NTI values.

### Differential enrichment of functional pathways across forest types

**Fig 5:**
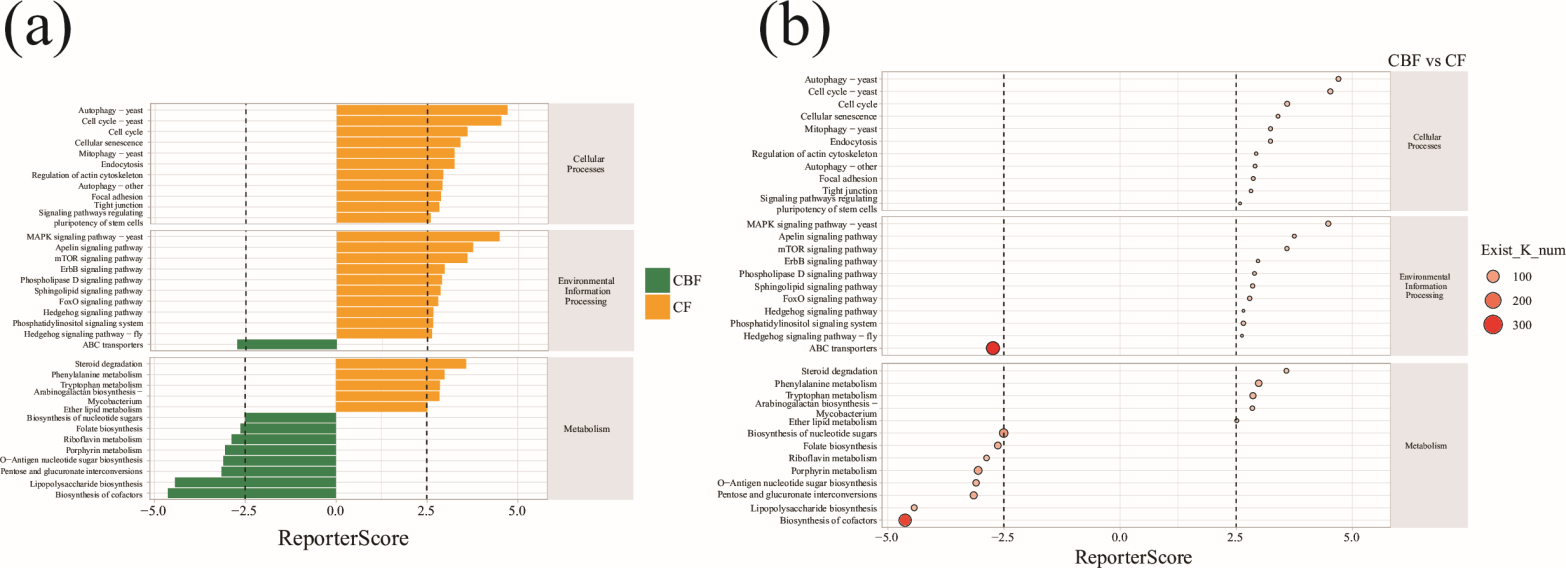
Functional pathway enrichment analysis. (**a**) Reporter score bar chart: the *y*-axis represents pathway categories, and the *x*-axis represents the Reporter score. This chart shows the differences in functional pathway enrichment between the coniferous forest community (CBF) and the conifer-broadleaf mixed forest community (CF). (**b**) Enrichment bubble chart: the *y*-axis represents pathway categories, and the *x*-axis represents the Reporter score. The bubble size indicates the number of KO terms detected for each pathway (Exist_K_num).

Functional enrichment analysis revealed distinct metabolic profiles between soil microbial communities in mixed conifer-broadleaf and coniferous forests. Communities in mixed forests showed significant enrichment in pathways involved in glycosylation, vitamin biosynthesis, and cellular repair, indicating elevated metabolic activity and enhanced capacity for cellular maintenance and environmental responsiveness (Fig. 5a). In contrast, microbial communities in coniferous forests were enriched in functions related to autophagy, cell cycle control, cellular senescence, mitophagy, endocytosis, cytoskeleton organization, MAPK signaling, mTOR signaling, and sphingolipid metabolism. This functional profile suggests a heightened stress response and adaptation to resource limitation (Fig. 5a). A bubble plot of enriched pathways further highlighted both the number and magnitude of these functional differences between forest types (Fig. 5b).

### Differential abundance of functional genes in carbon, nitrogen, phosphorus, and sulfur cycling

**Fig 6:**
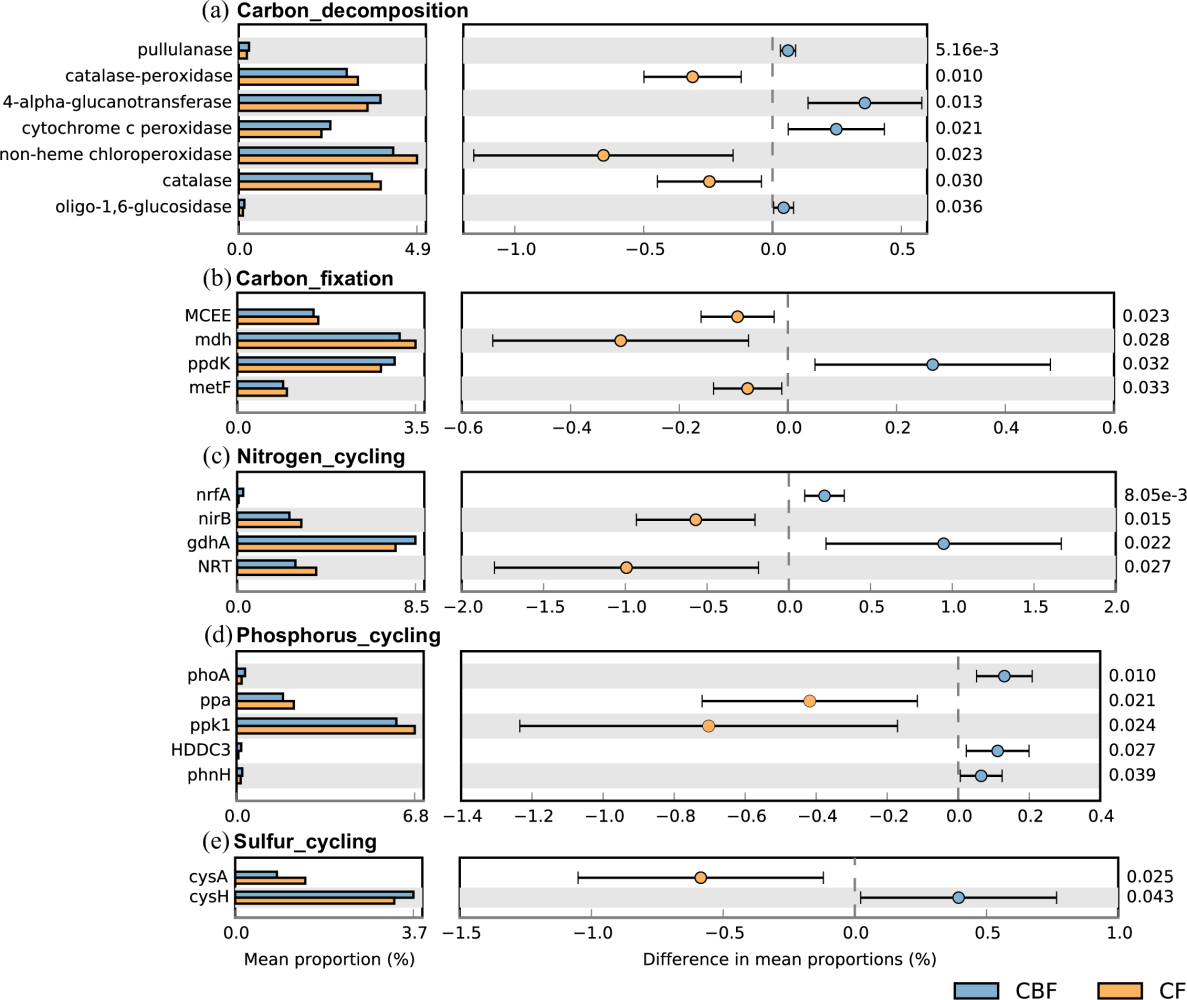
Genes that exhibit significant differences between mixed conifer-broadleaf forests and coniferous forests. (**a**) Carbon decomposition, (**b**) carbon fixation, (**c**) nitrogen cycling, (**d**) phosphorus cycling, and (**e**) sulfur cycling. In each panel, the left bars show mean relative abundance in each forest type, the middle section shows the proportion of differences within the 95% confidence interval, and the rightmost value is the corrected *P*-value.

Significant differences in the genetic potential for biogeochemical cycling were observed between forest types. Microbial communities in mixed conifer-broadleaf forest soils exhibited elevated abundances of genes encoding pullulanase, 4-alpha-glucanotransferase, cytochrome c peroxidase, and oligo-1,6-glucosidase—enzymes involved in polysaccharide and starch degradation, oxidative stress response, and carbon metabolism (Fig. 6a). This genetic profile suggests enhanced metabolic activity in carbon processing and energy acquisition. In contrast, coniferous forest soils were enriched in genes encoding catalase-peroxidase, non-heme chloroperoxidase, and catalase, indicating a heightened capacity for oxidative stress response and detoxification (Fig. 6a).

For central carbon metabolism, mixed forest soils showed significant enrichment in *ppdk* (pyruvate phosphate dikinase), a key enzyme in gluconeogenesis that converts pyruvate to phosphoenolpyruvate (Fig. 6b). Coniferous forest microbial communities, however, were enriched in *MCEE* (involved in amino acid and fatty acid metabolism), *mdh* (catalyzing malate-oxaloacetate conversion in the TCA cycle), and *metF* (supporting folate-mediated methylation and DNA synthesis), suggesting a focus on energy production and biosynthetic pathways (Fig. 6b).

Nitrogen cycling potential also differed markedly: mixed forest soils were enriched in *nrfA* (nitrate reductase) and *gdhA* (glutamate dehydrogenase), supporting nitrate reduction and nitrogen assimilation (Fig. 6c). Soils under coniferous forest displayed higher abundances of *nirB* (nitrite reductase) and nitrate transporter (*NRT*) genes, indicating adaptations toward denitrification and nitrate uptake (Fig. 6c).

Phosphorus metabolism genes such as *phoA* (alkaline phosphatase), *HDDC3* (hydrolase), and *phnH* (phosphoesterase) were more abundant in mixed forests, suggesting enhanced organic phosphorus mineralization (Fig. 6d). Conversely, coniferous forest communities were enriched in *ppa* (inorganic pyrophosphatase) and *ppk1* (polyphosphate kinase), reflecting adaptations in energy-efficient phosphate storage and stress response (Fig. 6d).

Sulfur cycling genes also exhibited forest-type specificity: *cysA* (cysteine transporter) was enriched in mixed forests, indicating efficient sulfur acquisition (Fig. 6e), while *cysH* (cysteine synthase) was more abundant in coniferous forests, suggesting enhanced cysteine biosynthesis and support for antioxidant production under stress (Fig. 6e).

## DISCUSSION

The bacterial communities in both forest types exhibited a characteristic long-tail distribution, with a small number of highly abundant taxa dominating the sequencing reads. Despite a marginally higher overall diversity in the conifer forest, no significant differences in α-diversity were detected between the abundant and rare subcommunities. The rare biosphere displayed significantly higher phylogenetic diversity, indicative of broader evolutionary origins and a potentially wider range of metabolic capabilities. A substantial compositional turnover was observed among rare taxa between the two forests, with a considerable number of OTUs showing significant enrichment or depletion in the mixed forest, highlighting their acute responsiveness to environmental variation (37, 38). Notably, despite their low relative abundance, rare taxa are likely to play a disproportionately large role in multi-nutrient cycling and the maintenance of ecosystem functions. These rare taxa contribute to biogeochemical cycles by performing specialized functions such as nitrogen fixation, denitrification, or the breakdown of recalcitrant organic compounds, processes that are crucial for nutrient transformation in nutrient-poor or fluctuating environments (39). They can also participate in the cycling of other elements, such as sulfur and phosphorus, through unique enzymatic pathways adapted to extreme conditions (40). Their ability to survive in nutrient-limited or hostile environments makes them key players in maintaining ecosystem stability under changing conditions.

In contrast, abundant taxa typically have broader metabolic capabilities and exhibit a wider niche range, enabling them to thrive in a variety of environmental conditions (41). These taxa often dominate key ecosystem processes such as organic matter decomposition, carbon cycling, and nutrient recycling under stable conditions (42). Their higher relative abundance means they play a pivotal role in maintaining the overall functioning of the ecosystem, especially in environments where resource availability is relatively stable. However, in more dynamic or extreme environments, abundant taxa may rely on rare taxa to support processes that require more specialized functions (38). These interactions between abundant and rare taxa ensure ecosystem stability and resilience, allowing ecosystems to maintain functionality across different environmental conditions.

Redundancy analysis identified SOM and pH as the primary drivers shaping both the total and abundant bacterial communities. In contrast, AP was the principal factor influencing rare taxa, indicating that rare species occupy narrower nutrient niches and exhibit a heightened sensitivity to phosphorus availability (43). The established relationship between pH and network complexity was evident here: the elevated pH and SOM in the mixed forest correlated with greater network connectivity and clustering, suggesting that alkaline, carbon-rich soils promote more complex and potentially stable microbial networks (44, 45). Conversely, declining pH and SOM lead to network fragmentation, which may undermine ecosystem resilience (46). These findings imply that management practices targeting soil pH and organic matter could be leveraged to influence microbial community structure and stability (47).

The soil’s physicochemical properties foster microbial diversity by creating a mosaic of microhabitats (48). Soil texture further modulates water retention and diffusive processes, generating micro-gradients of nutrients and oxygen that shape the microbial niche (49). This physical heterogeneity interacts with chemical gradients to facilitate the coexistence of generalists and specialists (50). Moving forward, integrating trait-based measures—such as metabolic breadth and stress tolerance—with occupancy-abundance relationships will be crucial to refine classifications of rarity and distinguish between conditionally rare, dormant, and endemic taxa (51, 52). A framework that synthesizes trait-based ecology with network and assembly theory will significantly enhance our ability to predict community responses to environmental change (53).

Co-occurrence network analysis further delineated the distinct roles of abundant and rare taxa. The network of the total community exhibited high connectivity and modularity. The abundant subnetwork, however, was simpler, with fewer nodes and connections, suggesting that dominant taxa engage in generalist interactions that form a stable structural backbone (54). Although the rare taxon network displayed low edge density, indicative of peripheral interactions, certain rare OTUs functioned as keystone hubs. These rare taxa, despite their low abundance, play critical roles in linking different microbial communities, facilitating nutrient cycling, and maintaining the stability of the overall network. For example, rare OTUs may contribute to specialized processes such as nitrogen fixation, sulfur oxidation, or the degradation of complex organic compounds, processes that are crucial for the overall nutrient balance and ecosystem resilience. These taxa often serve as essential genetic reservoirs, able to adapt rapidly to environmental changes, thus helping the community recover from disturbances (55). This pattern, corroborated by studies in alpine soils and lake sediments, positions rare taxa as critical genetic reservoirs within the community (56). The identification of a “small-world” architecture—where a few highly connected nodes bridge modules—highlights the disproportionate role of these rare keystone species and underscores the potential vulnerability of the network to their loss (57, 58).

Analysis of community assembly mechanisms revealed the dominance of stochastic processes across all community subsets. Ecological drift explained approximately 80% of the compositional variation in the total and rare communities, while dispersal limitation accounted for around 72% of the variation among abundant taxa. This finding aligns with reports from mountain forests where stochastic assembly prevails (59), though it contrasts with studies reporting deterministic control of rare taxa (60). Such discrepancies likely arise from differences in spatial scale, environmental heterogeneity, and taxonomic resolution (61). In our system, the small population sizes of rare taxa may heighten their vulnerability to ecological drift, while dispersal limitation likely shapes abundant taxa across heterogeneous microhabitats (62). The greater plant diversity and exudate heterogeneity in the mixed forest probably exacerbate niche differentiation and dispersal limitation, whereas the uniform conifer environment may amplify the role of drift (63). This interplay necessitates the separate consideration of abundance classes when inferring assembly rules.

The enhanced carbon and nitrogen metabolic pathways observed in the mixed forest align with experimental demonstrations that labile carbon exudates stimulate nitrogen turnover and activate previously dormant or rare microbial taxa (64). Conversely, the enrichment of stress-related and immune pathways in the conifer plantation suggests a community dominated by taxa adapted to the challenges of acidic, nutrient-depleted soils. The particular sensitivity of rare taxa to available phosphorus (AP) and potassium (AK) reinforces the concept that these organisms occupy narrow nutrient niches despite their pivotal role in multi-nutrient cycling (65). This is further supported by metagenomic studies indicating that rare bacterial diversity is a stronger predictor of carbon decomposition rates than the diversity of abundant taxa (66). The topological importance of rare taxa within our co-occurrence networks—exemplified by their roles as keystone hubs—parallels discoveries from alkaline lake sediments, where rare microbes form a persistent genetic reservoir and maintain network integrity (56). Furthermore, the overwhelming influence of stochastic processes (e.g., ecological drift, dispersal limitation) on community assembly echoes work in mountain forest ecosystems (59). The divergence from studies reporting deterministic control of rare assemblages underscores the profound context-dependency of these ecological rules, which are likely governed by spatial scale and environmental heterogeneity. Functional profiling illuminated a fundamental metabolic division between forest types, underpinned by a functional guild separation between abundant and rare taxa. In the conifer-broadleaf mixed forests, we noted a significant enrichment of genes encoding carbohydrate-active enzymes (e.g., pullulanase, 4-alpha-glucanotransferase) and central carbon assimilation proteins like pyruvate phosphate dikinase (ppdk). These genes are directly involved in the breakdown and assimilation of complex carbohydrates, which are critical for soil respiration, organic matter degradation, and carbon cycling in forest ecosystems. The presence of these genes suggests that rare and abundant taxa in mixed forests play essential roles in facilitating primary production and maintaining nutrient cycling by enabling the breakdown of organic materials into bioavailable carbon sources. Such genes, typically attributed to abundant generalist bacteria, are crucial for high-level ecosystem processes such as respiration and primary production, ensuring the forest soil’s vitality and long-term nutrient cycling (67, 68).

In contrast, coniferous soils were enriched for genes involved in heterotrophic energy metabolism (e.g., methylmalonyl-CoA epimerase, malate dehydrogenase), consistent with the resource-acquisition strategies of dominant taxa in nutrient-poor conditions (69). Most notably, the mixed forest exhibited a pronounced enrichment of specialized genetic determinants for niche-specific processes. These included *nrfA* (dissimilatory nitrite reduction to ammonium), phnH (organophosphonate degradation), and *phoA* (alkaline phosphatase production), which facilitate key biogeochemical transformations under specific nutrient conditions (70, 71). The carriage of such specialized genes within the rare biosphere, which possesses considerable genetic and functional redundancy, enables these taxa to execute critical steps in nitrogen, phosphorus, and sulfur cycling despite low abundances (72). This functional capacity, coupled with their seed-bank potential to rapidly respond to change, positions rare taxa as essential agents of ecosystem resilience (2).

This functional specialization was also evident in the coniferous forest, where the enrichment of *cysH*—a gene essential for assimilatory sulfate reduction into cysteine—suggests an adaptation to sulfur stress. This niche-specific pathway, often facilitated by rare bacteria, enhances community stability through functional redundancy during environmental fluctuations (73). Collectively, these results provide genomic evidence supporting the paradigm that abundant taxa sustain core ecosystem metabolism, while rare taxa provide critical, specialized catalytic functions (74). This synergistic partnership between abundance classes is a fundamental mechanism maintaining multi-nutrient cycling across contrasting forest biomes.

### Conclusion

This study elucidates the interdependent roles of plant diversity and soil chemistry in structuring soil bacterial communities across coniferous and mixed conifer-broadleaf forests. By integrating high-throughput sequencing, metagenomic functional profiling, co-occurrence network analysis, and null modeling of community assembly, we demonstrate that forest type acts as a primary determinant of soil edaphic conditions, with soil organic matter and pH serving as key predictors of total and abundant community structure and promoters of network complexity and stability. In contrast, available phosphorus and potassium exerted more subtle yet distinct selective pressures across abundance categories. Functionally, mixed forests were enriched for carbon and nitrogen metabolic pathways, while conifer plantations showed enhanced stress-related signaling and immune functions. Stochastic processes, particularly ecological drift, dominated community assembly in both forest types, although dispersal limitation emerged as a significant constraint for abundant taxa. Looking forward, future studies incorporating temporal dynamics and multi-omics approaches will be essential to unravel the causal mechanisms underlying plant-soil-microbe interactions and predict ecosystem responses to environmental change.

## Data Availability

All raw sequencing data have been deposited in the NCBI Sequence Read Archive (SRA) under BioProject accessions PRJNA1304032 and PRJNA1304125.

## SUPPLEMENTAL MATERIAL

Supplemental Material msphere.00627-25-s0001.docx Figure S1 and Table S1.

ASM does not own the copyrights to Supplemental Material that may be linked to, or accessed through, an article. The authors have granted ASM a non-exclusive, world-wide license to publish the Supplemental Material files. Please contact the corresponding author directly for reuse.
