# Supplementary material for: Differential assembly and functional roles of bacterial communities in coniferous and mixed conifer-broadleaf forest soils
Source: mSphere. 2026 Mar 6;11(3):e00627-25. doi: 10.1128/msphere.00627-25 (PMC13041531; doi:10.1128/msphere.00627-25)
Supplement: Supplemental Material — Figure S1 and Table S1. [file msphere.00627-25-s0001.docx]

**Differential Assembly and Functional Roles of Bacterial Communities in Coniferous and Mixed Conifer–Broadleaf Forest Soils**

Dexing Chen^1, 2^, Ziyang Zhang ^1, 2^, Shunfen Wang ^1, 2^, Wenhui Li ^1, 2^, Yimin He ^1, 2^, Wenyu Zhang^3^, Weiwei Sun^4^, Mingjiu Chen^4^, Shuangquan Zou^1, 2^*, Xin Qian^3^*

1 Fujian Colleges and Universities Engineering Research Institute of Conservation and Utilization of Natural Bioresources, College of Forestry, Fujian Agriculture and Forestry University, Fuzhou 350002, China

2 Key Laboratory of National Forestry and Grassland Administration for Orchid Conservation and Utilization at Colleage of Landscape Architecture, Fujian Agriculture and Forestry University, Fuzhou 350002, China

3 College of Forestry, Fujian Agriculture and Forestry University, Fuzhou 350002, China

4 Gufeng State-owned Forest Farm of Pingnan County, Pingnan 352300, China

* Corresponding author:

Shuangquan Zou, E-mail: zou@fafu.edu.cn;

Xin Qian, E-mail: qxxb2006@163.com

**
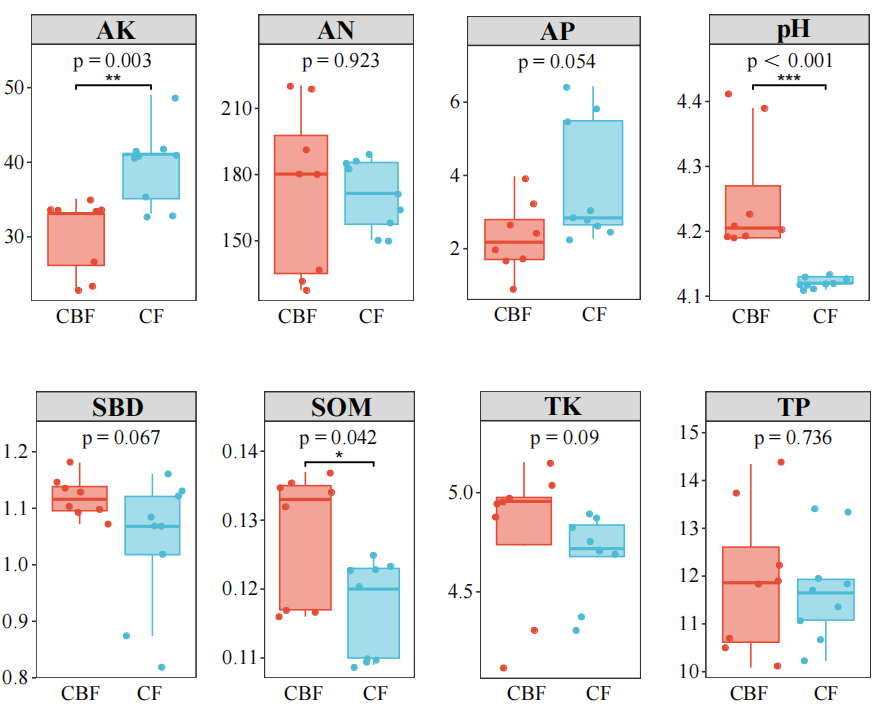
**

Figure S1. Differences in soil physicochemical proSoil physicochemical differences between forest types. Boxplots (with individual points) compare soil physicochemical properties between mixed conifer–broadleaf forest (CBF) and conifer forest (CF) sites, including available nitrogen (AN), available phosphorus (AP), exchangeable potassium (AK), soil pH, soil organic matter (SOM), soil bulk density (SBD), total potassium (TK), and total phosphorus (TP). P values are shown in each panel; significance is indicated by asterisks (*P < 0.05, **P < 0.01, ***P < 0.001).

Table S1. Co-occurrence network topology. Network properties of the total, abundant, and rare bacterial communities.

|  | **nodes** | **edges** | **positive.cor** | **negative.cor** | **average degree** | **average path length** | **diameter** | **density** | **clustering coefficient** |
| --- | --- | --- | --- | --- | --- | --- | --- | --- | --- |
| Total bacteria | 153 | 252 | 244 | 8 | 3.29 | 3.97 | 9.62 | 0.022 | 0.058 |
| Abundant | 87 | 105 | 104 | 1 | 2.41 | 3.69 | 9.56 | 0.028 | 0.028 |
| Rare | 97 | 84 | 83 | 1 | 1.73 | 5.86 | 16.03 | 0.018 | 0.027 |
